# Domain Adaptive Hand Pose Estimation Based on Self-Looping Adversarial Training Strategy

**DOI:** 10.3390/s22228843

**Published:** 2022-11-15

**Authors:** Rui Jin, Jianyu Yang

**Affiliations:** School of Rail Transportation, Soochow University, 8 Jixue Road, Xiangcheng District, Suzhou 215100, China

**Keywords:** hand pose estimation, adversarial training, domain adaptation

## Abstract

In recent years, with the development of deep learning methods, hand pose estimation based on monocular RGB images has made great progress. However, insufficient labeled training datasets remain an important bottleneck for hand pose estimation. Because synthetic datasets can acquire a large number of images with precise annotations, existing methods address this problem by using data from easily accessible synthetic datasets. Domain adaptation is a method for transferring knowledge from a labeled source domain to an unlabeled target domain. However, many domain adaptation methods fail to achieve good results in realistic datasets due to the domain gap. In this paper, we design a self-looping adversarial training strategy to reduce the domain gap between synthetic and realistic domains. Specifically, we use a multi-branch structure. Then, a new adversarial training strategy we designed for the regression task is introduced to reduce the size of the output space. As such, our model can reduce the domain gap and thus improve the prediction performance of the model. The experiments using H3D and STB datasets show that our method significantly outperforms state-of-the-art domain adaptive methods.

## 1. Introduction

Human hand poses are one of the long-standing research topics in computer vision. In recent decades, hand pose has had a wide range of applications in VR/AR, robot control [1], and human–machine interaction. Moreover, similar to human pose estimation for action recognition, 3D hand pose estimation can be further applied to gesture recognition and sign language recognition [2,3,4,5,6,7]. Despite the great success of applying deep neural networks to pose estimation tasks [8,9,10,11,12,13,14], the lack of well-labeled datasets has limited the development of powerful deep learning methods for hand pose estimation tasks. It is not an easy task to annotate real-world images because it is time-consuming and labor-intensive. To solve the problem of a lack of data, some researchers started to study synthetic datasets [15] after finding them easily accessible [16,17,18,19,20,21]. Therefore, making use of synthetic data, which is of high sufficiency, is the mainstream method to make up for the deficiency of training data in hand pose estimation.

However, there is a domain gap in skin texture and background between synthetic and real images, and this gap can affect the performance of the trained model [17,18,19,20,21]. To solve the domain gap problem, many methods have been proposed in existing works, for example, Mueller et al. [20] proposed a Cyclegan network, which aims to make synthetic images closer to realistic images in terms of background and texture. In the unlabeled target domain, in order to make use of the output of the teacher network, a mean-teacher model was proposed by Tarvainen et al. [22] for the guidance of the training of student network. During the domain shift, the noise of the pseudo labels would affect the training process of the model, which may lower the performance. As illustrated in the literature, the regression space of key point estimation is generally continuous. Therefore, the sparsity of the regression space was discovered by Jiang et al. [19] in a probabilistic sense. Then, a domain adaptive method was proposed in an unsupervised way, which is called RegDA. The RegDA method transforms the mini–max game between regressors to achieve the minimization of two opposite goals. Based on this, RegDA reduces the domain gap by adversarial training.

**Figure 1:**
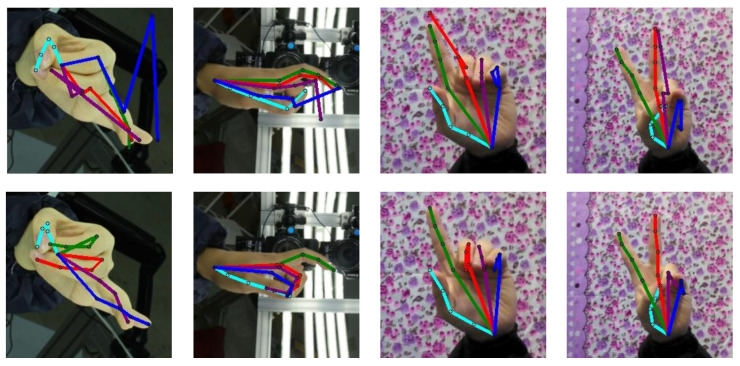
Some visualizations on the unlabeled target domains. The first row is the prediction results of the source-only model. The second row shows that the results of our model are more accurate. The colors indicate the estimated skeletons of different fingers.

To solve the issue caused by the large output space, both RegDA [19] and MarsDA [23] consider the sparsity of the regression space. This means that, if there is wrong prediction on the target domain, the distribution of the error predictions is not uniform in the pixel space. For an error predicted position of the finger joint, for example, the wrong position is always located at an adjacent fingertip or other key points. It is rarely located in the background. However, this is not absolute (see Figure 1), and when we only consider spatial locations with high probability and ignore locations with low probability, it will affect the accuracy and robustness of model prediction. The output space of the estimation model usually has a size of 64 × 64, and we can consider reducing the output space size such as 16 × 16. Inspired by this, we designed a new adversarial training strategy to reduce the output space of the regression task by adding a refinement module. With the reduced output space, we can effectively perform adversarial training to improve the prediction performance of the network and reduce the output fluctuations.

In this paper, we propose a new domain adaptive method for hand pose estimation. It can effectively reduce the domain gap and extract domain invariant features. We use the mean-teacher network to compose a multi-branch output model. We train the mean-teacher network on the synthetic data with labels. Furthermore, a set of pseudo labels are then generated for the unlabeled real-world data. However, the noise of the pseudo labels limits the accuracy of the model. To this end, we solve this problem by performing adversarial training with three branching networks. We add a refinement module to the student network to change the size of the output space to 16 × 16, thus assisting the model in adversarial training. The redesigned adversarial training strategy can effectively reduce the noise of pseudo labels. Our model is trained using accurate pseudo-labels, which ultimately results in better prediction accuracy.

We perform experiments on the H3D [24] and STB [25] datasets to evaluate the proposed method, and the results show that RegDA yields the best prediction results. The main contributions are as follows.

- We designed a new unsupervised domain adaptive model for hand pose estimation, which designed self-looping adversarial training strategy to bridge the gap between synthetic and real-world images.
- A new self-looping adversarial training strategy was designed to learn domain-invariant features more efficiently, which can lead to more accurate pseudo labels generated by the teacher network.
- Achieving state-of-the-art performance on H3D and STB real-world datasets demonstrates that self-looping adversarial training strategies can effectively reduce domain differences.

We organize the rest of this paper as follows. The related work is discussed in Section 2. The proposed method is then presented in Section 3. After that, experimental evaluation and analysis are performed in Section 4. Finally, Section 5 concludes the paper.

## 2. Related Work

### 2.1. Hand Pose Estimation

In recent decades, hand posture estimation has attracted the attention of many researchers, so there are very many innovations and applications [3,5,20,26,27,28,29,30,31]. Thanks to the rapid development of deep learning algorithms, hand pose estimation based on RGB images has become a popular research topic [3,32,33,34,35]. However, deep learning algorithms require many labeled data to train the model, so that a good model can be obtained. High-quality RGB hand pose datasets are very scarce, which restricted the development of pose estimation. Some researchers have started to find solutions. Spurr et al. [36] proposed a VAE-based “cross-modal variational model” that learns the shared latent space between different modalities. Wan et al. [37] proposed a network structure based on two generative networks with the goal of 3D hand pose estimation. The network consists of a variational autoencoder for hand pose and a generative adversarial network for deep image distribution modeling.

As the rendering technology has undergone significant development, synthetic datasets are widely used by researchers in order to assist in real-world hand pose estimation tasks. To enable the adaptation from synthetic datasets to the real-world unlabeled datasets, an end-to-end network was proposed by Dibra et al. [38]. A separated potential space was proposed by Yang et al. [39] to separate the image variations, e.g., the image background content and hand pose, which can be utilized to estimate hand pose and for image synthesis. There are several widely used benchmark datasets for testing hand pose estimation methods, including the Stereo Hand Pose Tracking Benchmark (STB) [25], the Rendered Hand Pose Dataset (RHD) [17], and the Hand-3D-Studio dataset (H3D) [24]. The proposed method is tested on these benchmarks and achieves state-of-the-art performance on H3D and STB with an accuracy of 81.3% and 82.4%.

### 2.2. Unsupervised Domain Adaptation

The unsupervised domain adaptation methods are designed to train a model in the unlabeled target domain from a labeled source domain. Then, the trained model on the unlabeled target domain performs well. Using the adversarial learning is the mainstream approach. The domain invariant features are learned by a feature extractor to fool a domain discriminator. Domain adaptation has many applications in areas such as semantic segmentation, classification tasks, and image recognition. Domain adaptation has many applications in areas such as semantic segmentation, classification tasks, and image recognition [9,40,41,42,43]. Li et al. [44] proposed an MMD-AAE framework that aligns the features extracted from multiple domains. Sankaranarayanan et al. [45] proposed a domain adaptive method which uses an adversarial training framework for weak segmentation. However, the relationship between images and 3D poses is nonlinear, so stronger constraints are required to effectively eliminate the domain gap. These above algorithms do not obtain the expected results when applied to the hand estimation task, and for this reason, a new domain adaptive algorithm was designed.

## 3. The Proposed Method

**Figure 2:**
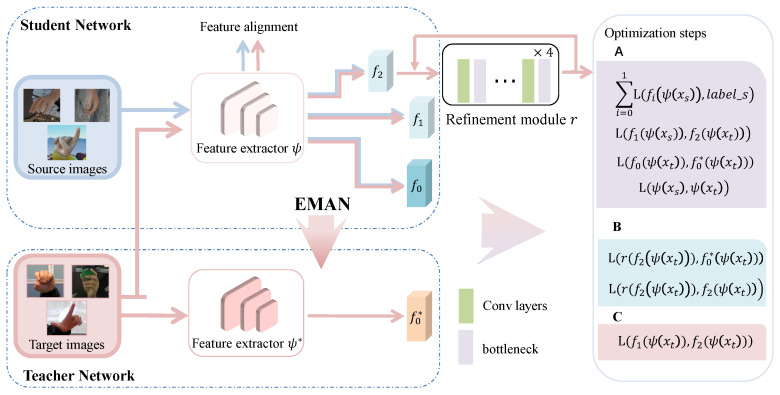
Our network is a student–teacher architecture, where the student network consists of a feature extractor, three regressors, and a refine module. The source and target images are fed into the student network, while the target images are fed into the teacher network. In addition, The teacher network also has three regressors, but the regressors $f_{2}^{*}$ and $f_{1}^{*}$, which correspond to the adversarial regressors and $f_{1}$ in the student network, are not used during training and testing, so we do not draw them.

In this section, we describe our unsupervised domain adaptation model in detail. Figure 2 shows the overview architecture. The acquired images of the unlabeled target domain are fed into the network, and finally, the network model outputs an accurate 2D hand pose. To this end, we design a student–teacher network. Both the teacher network and the student network are composed of a feature extractor and three regressors. The student network utilizes source and target domain data to train, while the teacher network generates pseudo-labels for the unlabeled target domain data. These pseudo labels are noisy due to the domain gap. Training the model directly with pseudo-labels will have an impact on the model prediction. To reduce this effect, we introduced an adversarial training strategy in the multi-branch structure.

### 3.1. Multi-Branch Domain Adaptation Module

The domain adaptation task contains data from two different domains, where the first domain is the source domain $X_{s}={\left\{x_{s}^{i}\right\}}_{i=1}^{N}$ with label $Y_{s}={\left\{y_{s}^{i}\right\}}_{i=1}^{N}$ and the second domain is the target domain $X_{t}={\left\{x_{t}^{i}\right\}}_{i=1}^{N}$. It worth noting that, the samples of the target domain are not the ground truth. The proposed student network includes a feature extractor, different regressors, and a refinement network. Both of the data in the source domain and the target domain are input into the model. The output of the teacher network is used as the pseudo label in the domain of the target. The teacher network and the output are denoted by symbols “*” and “∼”, respectively. The final prediction result is the output of the regressor when performing the testing phase.

Similarly to MarsDA [23], the student network is trained following the way of the standard supervised network in the training phase. The learning of the parameters θ of the student network is different from that of the parameters $\theta^{\prime }$ of the teacher network. The student network is learned using stochastic gradient descent (SGD), while the teacher network is not involved in gradient direction propagation. The exponential moving average normalization (EMAN) is employed to update the parameters $\theta^{\prime }$ of the teacher, i.e.,

(1) $$\theta_{t}^{\prime }=m\theta_{t-1}^{\prime }+\left(1-m\right)\theta_{t},$$

(2) $$\mu_{t}^{\prime }=m\mu_{t-1}^{\prime }+\left(1-m\right)\mu_{t},$$

(3) $${\sigma^{\prime }}_{t}^{2}=m{\sigma^{\prime }}_{t-1}^{2}+\left(1-m\right)\sigma_{t}^{2},$$

where μ is the mean of batch normalization (BN), and $\sigma^{2}$ is the variance of BN. *t* denotes the epoch number of training. The value of the momentum *m* is a number close to 1, i.e., 0.999.

For a better comparison with RegDA [19] and MarsDA [23], the loss between the ground truth and the estimated heatmaps is calculated by the Kullback–Leibler (KL) divergence. In the task of hand pose estimation, the same performance can be obtained for a model trained with KL divergence as for a model trained with MSE. First, a spatial probability distribution $P_{T}\left(H^{k}\right),k\in \left\{1,2,\ldots ,K\right\}$ is defined, which aims to normalize the heatmap of each key point $H^{k}\in R^{H\times W}$. *K* is the number of key points in the hand, K=21.

(4) $$P_{T}\left(H^{k}\right)=\frac{H^{k}}{\sum_{h=1}^{H}\sum_{w=1}^{W}{\left(H^{k}\right)}_{h,w}}.$$

Denoting Q(·) as the spatial softmax function:

(5) $$Q{\left(z\right)}_{h,w}=\frac{\exp(z_{h,w})}{\sum_{h=1}^{H}\sum_{w=1}^{W}\exp\left(z_{h,w}\right)},$$

We can use KL divergence to calculate the loss.

(6) $$L_{T}\left(H_{s},y_{s}\right)=\frac{1}{K}\sum_{k}^{K}KL\left(P_{T}\left(H_{y_{s}}^{k}\right)∥Q\left(H_{s}^{k}\right)\right),$$

where $H_{s}=f_{0}\left(\psi \left(x_{s}\right)\right)\in R^{K\times H\times W}$, ψ is the feature extractor, $f_{0}$ is the main regressor, and $H_{y_{s}}^{k}$ is the heatmap of each keypoint *k* in the label $y_{s}$.

Equation (6) represents the loss in the source domain between the heatmap predicted by the main regressor and the ground truth.

(7) $$L_{s}=L_{T}\left(H_{s},y_{s}\right).$$

We use the pseudo-label ${\tilde{y}}_{t}$ of the target domain instead of the ground truth, and thus calculate the loss function in the target domain.

(8) $$L_{T}\left(H_{t},{\tilde{y}}_{t}\right)=\frac{1}{K}\sum_{k}^{K}KL\left(P_{T}\left(H_{{\tilde{y}}_{t}}^{k}\right)∥Q\left(H_{t}^{k}\right)\right),$$

where ${\tilde{y}}_{t}=f_{0}^{*}\left(\psi^{*}\left(x_{t}\right)\right)$ is the pseudo-label. The output of regressor $f_{0}^{*}$ is the pseudo-labels.

To bridge the domain gap between the source and target domains, we apply a feature alignment loss to align the feature distributions between synthetic and real-world domains. Global feature alignment loss is introduced.

(9) $$L_{global}=L_{T}\left(F_{s},F_{t}\right).$$

This loss can mitigate the effect of noise on the pseudo-label at a certain level.

### 3.2. Self-Looping Adversarial Training

Since the data distributions of the source and target domains are different, it is a core problem of the domain adaptation task to measure the difference between the data distributions of these two domains. Currently, adversarial training is commonly used in domain adaptation tasks to align the data distribution of these domains.

Inspired by the latest theory [19,46], we designed an multi-branch adversarial training strategy. As shown in Figure 2, we use three regressors ($f_{0}$, $f_{1}$ and $f_{2}$) and a refinement module *r* to implement adversarial training. $f_{1}$ is the auxiliary regressor, and $f_{2}$ is the adversarial regressor.

The size of the output space of the hand pose estimation model is 64 × 64, while the classification model is much smaller than that. Therefore, we cannot directly apply the model for the classification task to the hand pose estimation task. For this problem, RegDA and MarsDA consider the sparsity of the regression space in the sense of probability, thus constraining the output space from a whole image space into a smaller one with only K key points. However, this method only considers the locations with higher probability in the output space, which is incomplete and affects the accuracy and robustness of the model prediction. To circumvent this problem, we propose a new adversarial training strategy that reduces the size of the output space to 16 × 16 using the refinement module.

The input of the refinement module is the output of the regressor $f_{2}$, which is denoted as $R_{t}$. As shown in Figure 2, we designed a self-feedback loop. We first made the output of the refinement module the K × 16 × 16 heatmaps. Then, we upsampled the output of the refinement module and used it to supervise the regressor $f_{2}$. As such, the gap between classification and regression may be bridged. We also proposed an error probability distribution to make the optimization of adversarial training easier. The distribution of error probability is generated using the pseudo labels in the target domain. That is, the ground error prediction, which is intended to make the distance from $f_{2}$ to the correct key points as far as possible. Hence, the optimization of $f_{2}$ is guided. The error probability distribution can be redesigned as follows:

(10) $$H_{F}\left(H^{k}\right)=I-R_{t},$$

where *I* is the matrix whose elements are all 1. Then, we can obtain $P_{F}\left(H^{k}\right)$:

(11) $$P_{F}\left(H^{k}\right)=\frac{H_{F}\left(H^{k}\right)}{\sum_{h=1}^{H}\sum_{w=1}^{W}{\left(H_{F}\left(H^{k}\right)\right)}_{h,w}}.$$

$P_{F}\left(H^{k}\right)$ represents the probability of the distribution of errors made by the model at different locations. Therefore, we convert the mini–max game of the two regressors to the minimization problem of two opposite objectives.

From the above, we need to keep the output of the refinement module away from the correct keypoint location. Then, the output of $f_{2}$ is supervised by the output of the refinement module via the self-feedback loop:

(12) $$L_{adv}=L_{T}\left(f_{2}\left(\psi \left(x_{t}\right)\right),R_{t}\right).$$

We concurrently supervised the output of the refinement module with the pseudo labels, i.e.,:

(13) $$L_{r}=L_{F}\left(R_{t},y_{t}^{\prime }\right),$$

where $R_{t}=r\left(H_{t}\right)$, $H_{t}=f_{0}\left(\psi \left(x_{t}\right)\right)$, $y_{t}^{\prime }$ is also the pseudo labels, and $y_{t}^{\prime }\in R^{K\times 16\times 16}$.

To reduce the difficulty of model training, we converted the max–min game in adversarial training into two minimization strategies. These two minimization strategies are defined as follows.

(14) $$L_{F}\left(M_{2},{\tilde{y}}_{t}\right)=\frac{1}{K}\sum_{k}^{K}KL\left(P_{F}\left(H_{{\tilde{y}}_{t}}^{k}\right)∥Q\left(M_{2}^{k}\right)\right),$$

(15) $$L_{T}\left(M_{2},M_{1}\right)=\frac{1}{K}\sum_{k}^{K}KL\left(P_{T}\left(H_{M_{1}}^{k}\right)∥Q\left(M_{2}^{k}\right)\right),$$

where $M_{1}=f_{1}\left(\psi \left(x_{t}\right)\right)$, $M_{2}=f_{2}\left(\psi \left(x_{t}\right)\right)$. They are the predicted results of two regressors $f_{1}$ and $f_{2}$.

### 3.3. Training Process

The purpose of the adversarial training strategy is to train the feature extractor ψ to deceive the adversarial regressor $f_{2}$, so that the feature extractor ψ can effectively learn domain invariant features. As with MarsDA, the final training steps are divided into three phases, namely A, B, and C. It is important to know that the loss functions in these three steps are optimized simultaneously in one framework.

Firstly, the feature extractor and the three regressors are trained using the source domain data, while the main regressor $f_{0}$ is trained using the pseudo-labels of the target domain. It should be noted that we minimize the loss functions of the adversarial regressors $f_{2}$ and $f_{1}$ on the source domain.

(16) $$\begin{array}{c} \min_{\psi ,f_{0},f_{1},f_{2}}E_{\left(x_{s},y_{s}\right)∼P}\left(L_{T}\left(f_{0}\left(\psi \left(x_{s}\right)\right),y_{s}\right)\right.\\ \;+L_{T}\left(f_{1}\left(\psi \left(x_{s}\right)\right),y_{s}\right)\\ \left.+\lambda_{1}L_{T}\left(f_{2}\left(\psi \left(x_{s}\right)\right),f_{1}\left(\psi \left(x_{s}\right)\right)\right)\right)\\ +\lambda_{2}E_{\left(x_{t},{\tilde{y}}_{t}\right)∼Q}\left(L_{T}\left(f_{0}\left(\psi \left(x_{t}\right)\right),{\tilde{y}}_{t}\right)\right)\\ +\lambda_{3}L_{T}\left(F_{s},F_{t}\right), \end{array}$$

where $\lambda_{1},\lambda_{2},\lambda_{3}$ are the weights to balance all losses.

Secondly, we minimize the losses of the adversarial regressor $f_{2}$ and the refinement module (Fix ψ, $f_{0}$ and $f_{1}$).

(17) $$\min_{f_{2}}\mu E_{\left(x_{t},{\tilde{y}}_{t}\right)∼Q}\left(\lambda_{4}L_{T}\left(f_{2}\left(\psi \left(x_{t}\right)\right),R_{t}\right)\right)+\lambda_{5}L_{F}\left(R_{t},{\tilde{y}}_{t}\right).$$

where $\lambda_{4},\lambda_{5}$ are the weights to balance all losses.

Thirdly, we train the feature extractor ψ to minimize the loss function between the regressors $f_{1}$ and $f_{2}$ over the target domain.

(18) $$\min_{\psi }\mu E_{\left(x_{t},{\tilde{y}}_{t}\right)∼Q}\left(L_{T}\left(f_{2}\left(\psi \left(x_{t}\right)\right),f_{1}\left(\psi \left(x_{t}\right)\right)\right)\right).$$

We keep repeating the above process to train the model and finally achieved the consistency of the data distribution in both domains.

## 4. Experiments

In the experimental section, we performed experiments using a synthetic dataset and two real-world datasets to validate our proposed method.

### 4.1. Datasets and Metrics

**RHD:** Rendered Hand Pose Dataset (RHD) [17] is a synthetic dataset with an image resolution of 320 × 320. The dataset is collected from 20 characters, where each character performs 39 different actions. This includes 4k training images as well as 3k testing images. All images are labeled with the locations of 2D and 3D keypoints. During the training process, we also cropped and resized the images to 256 × 256.

**H3D:** Hand-3D-Studio (H3D) [24] is a real-world dataset containing 22k images. It builds on hand poses performed by 10 people. Both sexes were represented and all the skin tones of the 10 people were different. According to RegDA, we used 3.2k images for testing, and used the rest for training. For the training process, cropped images were used with 512 × 512 resolution, which are provided by RegDA.

**STB:** Stereo Hand Pose Tracking Benchmark (STB) [25] is a dataset of the real-world including images with 640 × 480 resolution. This dataset has 18 k images, and 21 hand joint locations are collected as ground truths. The 18k images were divided into 15k images for training, and another 3k images for testing. In the training phase, the images are cropped and resized into 256 × 256.

We used the percentage of correct keypoints (PCK) as an evaluation metric. Specifically, we used PCK@0.05. That is to say, if the distance between the prediction and the ground truth is no more than a fraction α=0.05 of the size of an image, the prediction is regarded as a correct result. The average PCK of the 21 keypoints are calculated. At the same time, the PCK of different hand parts are also shown, e.g., the metacarpophalangeal joint (MCP), proximal interphalangeal joint (PIP), and distal interphalangeal joint (DIP), and the fingertip. RHD→H3D stands for the domain adaptation between the source dataset RHD and the target dataset H3D. RHD→STB stands for domain adaptation from the source dataset RHD to the target dataset STB. The image processing is implemented via Python.

### 4.2. Implementation Details

Resnet101 [8] is employed as the extractor ψ of features. We used two convolutional layers for regressors. There is a bottleneck block in the refinement module, followed by a convolutional layer. As with MarsDA [23], we cropped and resized the training images to 256 × 256. The whole model was trained for 100 epochs. The mini-batch SGD with a momentum was 0.9. The batch size was 32. We adjusted the learning rate by $l_{p}=l_{0}{\left(1+\alpha p\right)}^{-\beta }$. The p here denotes the step of the training. $l_{0}=0.1$, α=0.0001 and β=0.75. According to [47], we set the feature extractor learning rate to one tenth of the regressor. In the optimization phase, the weights of losses are separately set to $\lambda_{1}=6,\lambda_{3}=0.5,\lambda_{4}=0.8,\lambda_{5}=0.2$ and $\lambda_{2}=min\left(0.01*epoch,0.3\right)$.

### 4.3. Main Results

**Figure 3:**
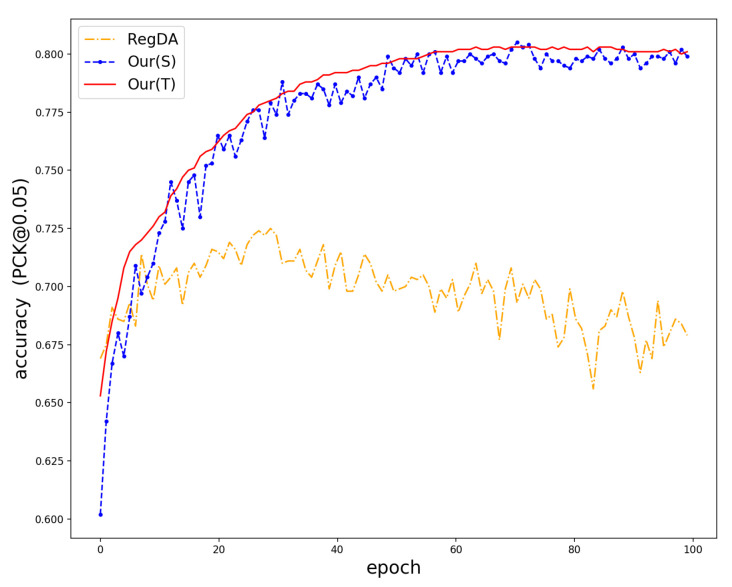
Accuracy of different models during training.

We compared the proposed method with some of the latest domain adaptive methods, and the experimental results are shown in Table 1 and Table 2. The experimental results show that the model directly trained with synthetic data does not achieve excellent performance. Although the teacher–student network improves the performance of the model, the performance improvement is limited due to the inaccurate pseudo-labeling. Methods such as MCD and DANN also struggled to obtain excellent performance in the hand pose estimation task due to the domain gap between the source and target domains. RegDA and MarsDA achieved better performance because they exploited the probabilistic sparsity of the model on the output space, allowing the model to efficiently learn domain invariant features. Compared with them, our method reduced the output space by self-looping adversarial training. Then, the gap between the source and target domains effectively were bridged, and the teacher network is allowed to generate accurate pseudo labels and help the model to be trained. The average accuracy is increased by more than 2% compared with MarsDA on the STB dataset. From Figure 3, this demonstrates that our model effectively improves the accuracy and robustness of the prediction.

**Figure 4:**
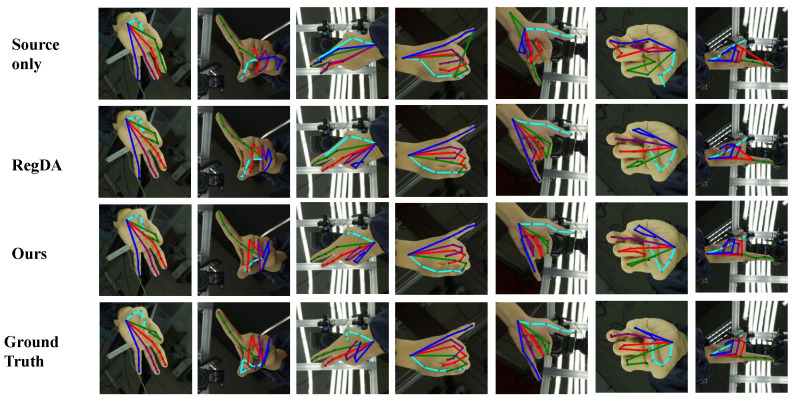
Qualitative results of different methods on the H3D dataset. The colors indicate the estimated skeletons of different fingers.

**Figure 5:**
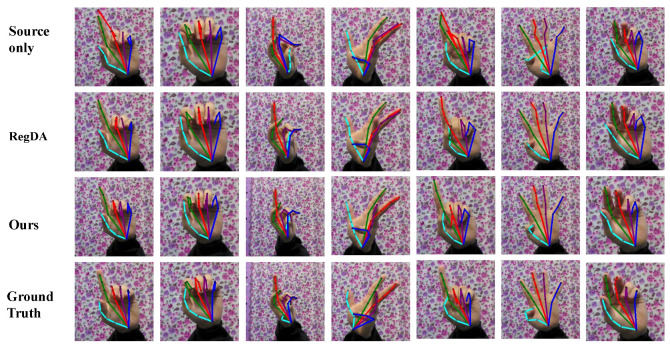
Qualitative results of different methods on the STB dataset.

To more visually demonstrate the superiority of the proposed method, we show some visualization results in Figure 4 and Figure 5. The proposed method is compared with other methods in the figures. From the figures, we can see that our method can obtain more accurate prediction results, while correcting the keypoints that other methods incorrectly predict.

### 4.4. Ablation Study

We conducted ablation experiments in the H3D dataset, which were used to investigate the contribution of different modules in the proposed model. The specific experimental results are shown in Table 3. The “source only” in the table refers to the model trained directly using the source domain data. “RD” refers to the RegDA network. “MT” refers to the mean-teacher network. “SAT” stands for the self-looping adversarial training strategy. “FA” refers to feature alignment. As can be seen, the new adversarial training strategy can effectively improve performance and bring 2.2% PCK improvement over the RegDA. The mean-teacher network gives a 3.8% performance improvement to the model, and feature alignment gives another 2.6% performance improvement. The self-looping adversarial strategy brings 1.6% PCK improvement. The final experimental results verify the superiority of the proposed method, which can obtain a PCK gain of 19.3% over the baseline. From the results, we can see that the method with SAT+MT+FA obtains the best performance of 81.3% in accuracy. This result indicates that the proposed self-looping adversarial training strategy significantly improves the result of hand pose estimation, which validates the effectiveness of this strategy. Furthermore, the feature alignment can also further improve the performance of the network.

We also conducted an ablation study to illustrate how different methods of adversarial training influences adaptation. Table 4 shows the results. The first row is RegDA, which minimizes two opposite goals separately. The second row is MarsDA, which optimizes the RegDA method. These are both trained by considering the sparsity of the output space in terms of probability. The last row is our method, where we reduce the size of the output space and thus perform the adversarial training. Our proposed method outperforms the first two methods to a large extent.

**Figure 6:**
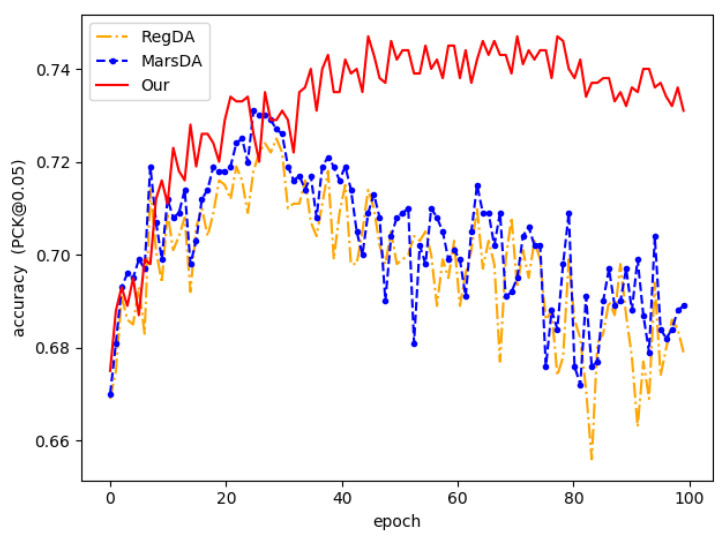
Accuracy of different adversarial training strategies during training.

**Figure 7:**
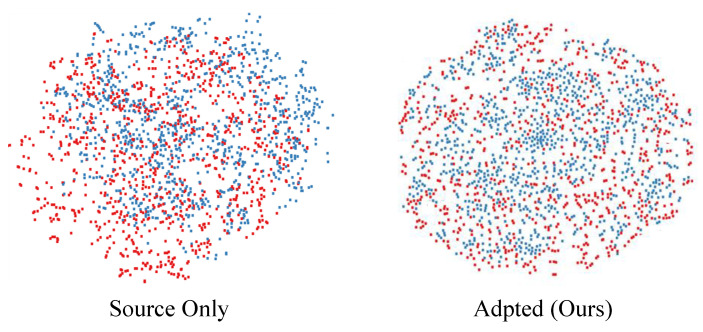
We randomly selected 1920 samples of synthetic and real-world images and used t-SNE [50] to visualize the features learned by the source-only model and our MarsDA model, respectively. The red points are samples in the source domain while the blue points are samples in the target domain.

The training process is visualized in Figure 6. For RegDA, we can see that RegDA, like several other domain adaptation methods, suffers from the problem that there is a significant drop in accuracy as the training epoch increases. Marsda is the same as RegDA in that it considers the sparsity of the regression space in the sense of probability. Its accuracy, although improved, still suffers from the large fluctuation of the network output. However, this does not occur in our method. This demonstrates that our model effectively converts the mini–max game between two regressors into the minimization of two opposite goals. From Figure 3, we can see the training processes of different methods. It is shown that our method outperforms it in terms of accuracy and stability. In order to more effectively demonstrate the robustness of the model, we used 10 epochs as a group to calculate the variance, and the results are shown in Table 4. As can be seen from the table, the variance calculated by Marsda and Regda in 50–100 epochs has a very large fluctuation, while the proposed method differs from them in that it has a very small fluctuation. More specifically, the variance values calculated by the proposed method in 50–100 epochs are within 0.01, while the other methods are above 0.4. Compared to the student network, the teacher network is much more stable than the student network because the teacher network is a temporal aggregation of the student network. To better demonstrate the distribution of features learned by the proposed model, we used t-sne [50] to visualize the features in the source and target domains extracted by the feature extractor. The results are shown in Figure 7, from which we can see that the proposed domain adaptive model can effectively reduce the domain differences and thus obtain better prediction performance.

## 5. Discussions

From the experimental results on different datasets, we can find that the colors will not affect the effectiveness of the proposed method. Meanwhile, it should be noted that, although the colors in the datasets are a little different, their difference is not significant. Since the data were captured in the lab with stable lighting, the results are stable under different illuminations. If there is an extreme illumination condition, the result depends on the effectiveness of hand detection results. Of course, the distance between the camera and the hand will influence the performance due to the change in the resolution of the hand. We conduct the experiments on the NVIDIA 3090 GPU to run the deep learning algorithms. The proposed method can be used for a robot, since the resources needed for inference are much less than that of training and the inference time is fast. The proposed method is robust with different backgrounds. From the figures of experimental results, we can find that the backgrounds of the images are significantly different, and the proposed method performs well with its promised results.

## 6. Conclusions

In this paper, a new unsupervised domain adaptation method is proposed for hand pose estimation. A self-looping adversarial training strategy is designed for knowledge transfer between the synthetic source domain and the real-world target domain. In the adversarial module, we reduce the size of the regression space, thus effectively converting the minimax game of the two regressors to a minimization problem between the two opposite goals. Thereby, the issue of the noise in pseudo labels at the training phase can be mitigated. Meanwhile, the data distribution between the synthetic and real-world domains are aligned. Extensive experiments on two benchmark datasets show the effectiveness of our approach. The main contributions including three main aspects. (1) We designed a new unsupervised domain adaptive model for hand pose estimation, which designs a self-looping adversarial training strategy to bridge the gap between synthetic and real-world images. (2) A new self-looping adversarial training strategy was designed to more efficiently learn domain-invariant features, which can lead to more accurate pseudo labels generated by the teacher network. (3) Achieving state-of-the-art performance on H3D and STB real-world datasets demonstrates that self-looping adversarial training strategies can effectively reduce domain differences.

## Figures and Tables

**Table 1:** Results on the task RHD→H3D. The last row (oracle) denotes the results of training with target domain labels.

Method	MCP	PIP	DIP	Fingertip	Avg
Res101 [8]	67.4	64.2	63.3	54.8	61.8
MCD [46]	59.1	56.1	54.7	46.9	54.6
DD [48]	72.7	69.6	66.2	54.4	65.2
DANN [47]	67.3	62.6	60.9	51.2	60.6
Cyclegan [49]	63.8	63.6	61.3	53.5	60.1
Mean-teacher [22]	72.6	71.2	67.1	59.4	66.8
RegDA [19]	79.6	54.4	71.2	62.9	72.5
MarsDA [23]	87.7	85.8	80.7	70.1	80.6
**Our**	**87.2**	**86.2**	**80.8**	**72.5**	**81.3**
Oracle	97.7	97.2	95.7	92.5	95.8

**Table 2:** Results on the task RHD→STB. The last row (oracle) denotes the results of training with target domain labels. The best performance is achieved by our MarsDA.

Method	MCP	PIP	DIP	Fingertip	Avg
Res101 [8]	67.6	65.4	65.9	59.9	63.1
MCD [46]	58.1	56.3	55.4	46.7	54.9
DD [48]	62.5	70.1	68.5	71.9	68.4
DANN [47]	68.1	64.1	65.1	59.2	63.1
Cyclegan [49]	58.2	58.5	57.9	58.9	58.1
Mean-teacher [22]	68.9	71.2	69.7	67.2	68.9
RegDA [19]	67.4	79.6	75.4	73.8	73.6
MarsDA [23]	75.7	84.7	81.2	83.5	80.2
**Our**	**79.8**	**89.1**	**84.9**	**76.8**	**82.4**
Oracle	93.9	93.0	93.8	94.4	93.4

**Table 3:** Ablation study results of the proposed model.

Method	MCP	PIP	DIP	Fingertip	Avg
Source only	67.4	64.2	63.3	54.8	61.8
+RD	79.6	74.4	71.2	62.9	72.5
+SAT	82.5	77.2	73.5	63.4	74.7
+RD+MT	82.6	82.2	79.1	67.4	76.3
+RD+MT+FA	85.4	84.6	79.9	67.6	78.9
+SAT+MT+FA	87.2	86.2	80.8	72.5	81.3

**Table 4:** Model Robustness Analysis.

Method	0–10	10–20	20–30	30–40	40–50	50–60	60–70	70–80	80–90	90–100
RegDA	1.471	0.367	0.247	0.405	0.376	0.196	0.726	1.087	1.432	0.450
MarsDA	1.578	0.378	0.216	0.196	0.659	0.714	0.565	0.848	1.188	0.594
Our	1.308	0.251	0.194	0.346	0.162	0.064	0.084	0.089	0.065	0.070

## Data Availability

Not applicable.
